# Nitrogen and phosphorus removal from anaerobic baffled reactor effluent using *Lemna minor* and fertiliser value of the biomass for ryegrass production

**DOI:** 10.1007/s10661-025-14592-4

**Published:** 2025-10-09

**Authors:** Pardon Muchaonyerwa, Adeshola A. Oyawoye, Alfred O. Odindo

**Affiliations:** School of Agricultural, Earth, and Environmental Sciences, University of KwaZulu-Natal, Private Bag X01, Scottsville, Durban, 3209 South Africa

**Keywords:** Biomass, Effluent, Fertiliser value, Nutrient removal, Inoculation density

## Abstract

Effluents from decentralised wastewater systems, using an anaerobic baffled reactor (ABR), have high concentrations of nitrogen (N) unsuitable for safe disposal. The study investigated the effects of duckweed (*Lemna minor*) density and effluent dilution on N removal and biomass accumulation, over 14 days. The duckweed biomass was tested as a fertiliser for ryegrass (*Lolium perenne*) at (i) 200 kg N ha^−1^ (DWN), (ii) 80 kg P ha^−1^ (DWP), (iii) DWN with mineral P to 80 kg P ha^−1^ (DWN + P), compared with (iv) inorganic N fertiliser and two negative controls with (v) P and potassium (K), and (vi) K only. Biomass and N (11–56 mg L^−1^) removal increased with effluent dilution and higher density. The 1:3 dilution (effluent: water) and chemical fertiliser (CF) had higher biomass than other treatments, except 1:1 at 800 g m^−2^. The treatments had similar percentage N removal (> 79%), except the 3:1 with 400 g m^−2^ duckweed (73%). Duckweed treatments had higher ryegrass dry matter, and N and P uptake, than the controls without N. The DWP and inorganic fertiliser with N had similar ryegrass dry matter and N uptake, which were higher than for DWN and DWN + P. The findings showed that duckweed, cultured at 600–800 g m^−2^, could efficiently remove N from diluted ABR effluent for safe disposal, and that duckweed biomass increases ryegrass N uptake and dry matter to similar levels as inorganic N fertiliser, especially when applied to meet P requirements.

## Introduction

Centralised technologies for managing human waste and wastewater are mostly not feasible in areas with rugged and hilly terrain on the periphery of urban settlements (Hudson, 2011). The decentralised wastewater treatment system (DEWATs) that uses anaerobic baffled reactors (ABR) to treat domestic wastewater could be appropriate in these areas. The series of hanging and standing (vertical) baffles of the ABR force wastewater to flow up and down, through a series of compartments, as it passes from the inlet to the outlet (Morel & Diener, 2006; Sasse, 1998). Although the ABR is efficient for the removal of organic material and suspended solids (Gutterer et al., 2009), the resulting effluent contains high ammonium-N (20–50 mg L^−1^) and orthophosphate-P (5 mg L^−1^) (Collivignarelli et al., 1990; Foxon et al., 2007) making it unsuitable for disposal into surface water. Aquatic plants offer an attractive solution to remove these nutrients from the effluent.

Floating aquatic plants (e.g. duckweed and water hyacinth) can improve water quality by regulating oxygen balance and accumulating nutrients and heavy metals. Different species of duckweed (*Spirodela* spp*.*, *Wolffia* spp*.*, and *Lemna* spp*.*) have been tested for the treatment of varying wastewater types with 50–95% treatment efficiency (Muradov et al., 2014; Ozengin & Elmaci, 2007), with the majority of the studies being conducted with swine effluent (Chikuvire et al., 2018a; Ge et al., 2012; Muradov et al., 2014; Xu & Shen, 2011; Zhao et al., 2014).

Muradov et al. (2014) reported that duckweed (*Landoltia punctata*) removed N at 3.0 mg L^−1^ day^−1^, and P at 0.15–0.38 mg L^−1^ day^−1^, which resulted in up to 87% N and up to 45% P removed from the dilute swine effluent. In addition to effluent concentration, harvesting frequency has been shown to be essential in terms of biomass accumulation of duckweed and N and P removal from effluents.

Xu and Shen (2011) reported that *Spirodela oligorrhiza,* grown on swine effluent, accumulated biomass at a rate of 1.93, 1.43, and 0.88 g m^−2^ day^−1^, when harvested twice per week, once per week, and once in two weeks, respectively. The authors reported 84–100% ammonium-N and 24–53% P removal from swine effluent after two weeks. Chikuvire et al. (2018a) reported *Wolffia arrhiza* biomass ranging from 0.88 to 1.83 g m^−2^ day^−1^ with > 98% ammonium N and > 96% P removed in 7 and 14 days, respectively. In general, effectiveness of wastewater treatment with duckweed is mainly dependent on wastewater type, duckweed species, and inoculation density, and available nutrients in the medium. Ge et al. (2012) reported that *Lemna minor* grown on swine effluent had an average biomass accumulation rate of 3.5 g m^−2^ day^−1^, and removal rate of 3.15 mg L^−1^ day^−1^ for N over an 18-day period. Based on similar pH, ammonium-N, and orthophosphate-P content of the 4% swine effluent used by Xu and Shen (2011) and the ABR effluent studied by Foxon et al. (2007), studies with swine effluent may, therefore, be good models for ABR effluents.

The most prevalent duckweed species in South Africa are *W. arrhiza* and *Lemna minor* (Chikuvire, 2018). Biomass accumulation of *L. minor* on ABR effluent and its effects on the removal of nitrogen and phosphorus are not understood. The pilot DEWATS system at Newlands-Mashu, South Africa, produces ABR effluent with pH 6.5, 20–50 mg L^−1^ ammonium-N (Foxon et al., 2007), making it unsafe for disposal into water bodies (Department of Water Affairs, 2013). Removal of these nutrients with duckweed could make it safe for disposal while generating biomass that could be a beneficial source of N and P for crops.

Several articles in the literature have shown that incorporation of high-quality plant residue improves soil nutrient availability (Partey et al., 2014; Tilley et al., 2014). The high-quality residues have high N and low tannin, lignin, and polyphenol concentrations (Palm et al., 2001). The high-quality residues can be used as nutrient sources without further addition of N fertilisers (Palm et al., 2001). The C:N ratio in aquatic plants such as duckweed (10:1) (Meyers & Doose, 1999) is lower than most terrestrial crops (higher than 20:1) (Stewart, 2010). The high N, low C:N, and lack of cellulose and lignin in aquatic plants, when compared with their terrestrial counterparts (Meyers & Doose, 1999), could result in rapid decomposition (Partey et al., 2014). Information on the use of duckweed dry matter as a nutrient source for plants is limited in the literature. Average tissue of *L. minor* grown on swine effluent had up to 4.58% N, and 0.67% P, suggesting that it can rapidly decompose and mineralise the N. If applied as a fertiliser based on N requirements, the duckweed biomass would not supply sufficient P. Applying the biomass as a source of P would supply excess N. It may, however, be essential to augment the P content in the duckweed by adding inorganic P fertiliser to make up for the difference in P. The concentrations of N and P in the tissue of L. minor grown on ABR effluent and the value of the biomass as a source of these nutrients are not known, in order to make decisions on co-application with inorganic fertilisers.

We hypothesised that increasing density of duckweed (*L. minor*) with dilution of ABR effluent would increase biomass accumulation and reduce N for safe disposal. It was further hypothesised that the *L. minor* biomass provides sufficient nutrients to sustain growth of perennial ryegrass. This study investigated the effect of inoculation density of duckweed (*L. minor)* and dilution of ABR effluent on removal of N and biomass accumulation, and the effects of the duckweed biomass on N and P uptake and dry matter yield of perennial ryegrass.

## Materials and methods

The experiments for this study were carried out in the growing tunnel at the Newlands-Mashu research site (29°58′S and 30°57′E), Durban, South Africa.

### Removal of nitrogen and phosphorus

#### Collection and analysis of ABR effluent

The ABR effluent was collected from the decentralised wastewater treatment (DEWATS) plant in the Newlands-Mashu research site, KwaZulu-Natal, and analysed for ammonium-N, nitrate–N and orthophosphate-P using the Merck® Spectroquant photometer. Electrical conductivity (EC) was measured with the sensION™ + MM150 portable multimeter (Hach® Company, Colorado, USA), according to Standard Methods for the Examination of Water and Wastewater (American Public Health Association 2005).

#### Duckweed cultures

The duckweed (*L. minor*) was collected at Ashburton (29°40′S; 30°27′E) in Pietermaritzburg, KwaZulu-Natal, and pre-conditioned in ABR effluent diluted with tap water at a 1:1 ratio for 5 days. The tap water used had pH 6.22, EC of 248 µS cm^−1^, and had undetectable levels of ammonium- and nitrate–N and orthophosphate-P. An Omnia® fertiliser solution, with 8.4 mg ammonium-N L^−1^, 3.6 mg nitrate N L^−1^, and 4.1 mg orthophosphate L^−1^, was used as a contamination control. The growth of the duckweed was not lowered by the diluted ABR effluent when compared to the fertiliser solution.

#### Experimental procedure

The experiment was a 3 × 5 factorial arrangement in a split-plot design with three replications. The main plot factor was inoculation density of duckweed, with ABR effluent concentration (dilution) as the subplot. The densities were 400, 600, and 800 g m^−2^ (fresh weight with average water content of 95%), which translated to 20, 30, and 40 g m^−2^, respectively, dry weight. The wide range of duckweed densities was selected in order to observe the optimum density that allows for biomass accumulation and nutrient removal from the effluent. The biomass had 4.1% N and 0.4% P; the densities translated to additions of 0.82, 1.23, and 1.65 g N m^−2^, and 0.08, 0.12, and 0.16 g P m^−2^. The ABR effluent treatments used were (i) undiluted effluent and effluent:tap water ratios of (ii) 3:1, (iii) 1:1, and (iv) 1:3, with (v) hydroponic solution as a control. The ABR effluent (63.1 mg ammonium-N L^−1^) was diluted to come up with an optimum dilution for growth of the duckweed and rapid nutrient removal, and to ensure that the ammonia-N content was lower than 60 mg N L^−1^ in order to avoid ammonia toxicity (Chikuvire et al., 2018b). Conversely, low nutrient concentrations would not support duckweed growth. The chemical fertiliser/hydroponic solution (CF), made by dissolution of a combination of calcium nitrate and a “New Natgrow” product, supplied by FlowGrow Hydroponics, in tap water, had 111, 17, 131, 97.5, 8.7, and 38.4 g kg^−1^ of N, P, potassium (K), calcium (Ca), magnesium (Mg), and sulphur (S), respectively. The characteristics of the different dilutions of effluent and hydroponic solution used in the study are presented in Table 1. Except for nitrate–N and pH, the effluent and its dilutions had higher levels of all parameters than the hydroponic solution. Concentrations of ammonium-N in the treatments were higher than the South African threshold of 3 mg N L^−1^, while those of orthophosphate-P were at or lower than the threshold of 10 mg P L^−1^ (Department of Water Affairs, 2013) at the commencement of the experiment. As a result, the study focussed on the removal of N and not P.

**Table 1:** Physico-chemical properties of different effluent dilutions and hydroponic solution used

Parameters	ABR	3:1	1:1	1:3	CF
pH	7.86	7.76	7.72	7.67	7.71
Electrical conductivity (dS m^−1^)	1.33	1.01	0.77	0.54	0.41
Ammonium N (mg L^−1^)	63.1	47.3	31.6	15.8	8.4
Nitrate N (mg L^−1^)	0.30	0.225	0.15	0.075	3.60
Orthophosphate P (mg L^−1^)	11.5	8.63	5.75	2.88	4.10

*CF*, chemical fertiliser/hydroponic solution; *ABR*, undiluted ABR effluent; 3:1, 1:1, and 1:3 are ratios of effluent, tap water. South African thresholds = 3 mg ammonium-N L^−1^; 15 mg nitrate–N L^−1^ and 10 mg orthophosphate-P L^−1^ (Department of Water Affairs, 2013)

Four litres of each solution were put in a plastic container with 0.0625 m^2^ surface area and 0.012 m depth, and the duckweed was added at the appropriate density before culturing for 14 days in a tunnel, with maximum and minimum air temperatures of 34 and 19 °C, respectively, and 65 to 80% relative humidity. A 20 mL sample was collected after 7 and 14 days, and replaced with 20 mL tap water (at 7 days). Additional tap water was added to address water losses through evapotranspiration (decline in solution level). The solutions were analysed for pH, EC, and ammonium- and nitrate–N. Percentage nutrient removal was calculated. The duckweed was harvested after 14 days, dried at 60 °C for three days, and weighed. Dry matter accumulation was calculated by subtracting the biomass added as inoculation.

### Fertiliser value of duckweed biomass

#### Duckweed biomass used

The duckweed (*L. minor*) grown at 600 g m^−2^ in a plastic tank with 1:1 ratio of ABR effluent:water, which was found to be the best in removing N, had high biomass production with high N and P concentrations. After 14 days, the duckweed biomass was dried at 60 °C for 72 h (705 g dry matter), and analysed for tissue N using the Leco TruMac CNS autoanalyser, and total P and K using inductively coupled plasma–atomic emission spectroscopy (ICP–AES) after digestion with nitric acid. The duckweed tissue contained 31.3 g N kg^−1^, 5.0 g P kg^−1^, and 14.5 g K kg^−1^, which informed the amount of N and P needed in the treatments.

#### Soil used

The soil used in this study was from the 0–30 cm depth of an arable field at KwaDinababuko, close to Hillcrest in Durban, South Africa. The soil was classified as the Cartref soil form (Soil Classification Working Group, 2018) and Typic Haplaquept (Soil Survey Staff, 2014). The soil was air-dried and ground to pass a 2 mm sieve before analysis. Soil particle size distribution was determined using the pipette method, while pH was determined in 1 M KCl at a 1:2.5 (soil:solution) ratio, using a combination glass electrode (The Non-Affiliated Soil Analysis Work Committee, 1990). Soil total C concentration was analysed using the automated Dumas dry combustion method, using a LECO CNS 2000 (Leco Corporation, MI, USA). Ambic-2 extracting solution, containing 0.25 M NH_4_CO_3_ + 0.01 M Na_2_EDTA + 0.01 M NH_4_F + 0.05 g L^−1^ Superfloc (N100), at pH 8, was used to extract P and K. Phosphorus was determined using the molybdenum blue procedure (Murphy & Riley, 1962). Potassium, Ca, and Mg were determined by atomic absorption spectroscopy after extraction with ammonium acetate (Manson & Roberts, 2000). The soil used had 110 g clay kg^−1^ (11%), pH 4.0, of 5 g C kg^−1^, and 0.7 mg P kg^−1^. Exchangeable K, Ca, and Mg were 0.02, 0.52, and 0.32 cmol_c_ kg^−1^.

#### Experimental procedure

The pot experiment was conducted in the same tunnel used for removal of N and P from ABR effluent. The experiment was set up in a randomised complete block design with five replicates. Two kilograms (2 kg) of Cartref soil was packed in 2-L non-draining plastic pots. The fertiliser materials (including duckweed) were added to supply N and P at recommended rates for perennial ryegrass based on soil analysis. The first three treatments were duckweed biomass applied at (i) 200 kg N ha^−1^ (DWN), (ii) 80 kg P ha^−1^ (DWP), which invariably supplied 417 kg N ha^−1^; (iii) DWN augmented with mineral P at 80 kg P ha^−1^** (**DWN + P). The mineral P, as sodium dihydrogen phosphate (NaH_2_PO_4_:25.83% P), was added to correct for possible P deficiencies in the DWN treatment. Three control treatments were all amended with 30 kg K ha^−1^, while N and P were applied at 200 and 80 kg N and P ha^−1^, respectively, using inorganic fertilisers as (iv) N and P, (v) P and (vi) negative control. The inorganic N was applied in the form of urea, while K was applied as KCl. The duckweed was pre-incubated for two weeks before planting perennial ryegrass, while inorganic fertilisers were added at planting. Chikuvire et al. (2018b) indicated that maximum mineralisation of N to ammonium-N in duckweed (*Wolffia* spp.) biomass had occurred within two weeks, and the highest nitrate–N was released in six-to-eight weeks of incubation, while Chikuvire (2018) found that pre-incubation for 28 days significantly increased Swiss Chard yield in an 8-week pot experiment. As such, more nitrification would occur during the duration of the pot experiment (about 8 weeks: 61 days) in the current study, making N available for the crop.

Perennial ryegrass (*Lolium perenne* L., cultivar Bronsyn) was planted at double the recommended rate (40 kg ha^−1^) in the pots on the 16th of November 2016, and the experiment was terminated on the 16th of January 2017 (after 60 days). Ryegrass was harvested periodically when it grew to a height of 20 cm, and harvested plants were cut at 5 cm above the soil surface. At each harvest, the biomass was dried at 60 °C for 72 h. Soil samples were collected from all pots after the last harvest of the ryegrass, air dried, sieved and analysed for total C and N, Ambic-extractable P and pH in KCl.

Histograms were used to test the normality of distribution, while Bartlett’s test was used to test for homogeneity of variances of the data before subjecting them to analysis of variance (ANOVA). Data from the experiment for recovery of N and P from ABR effluent were subjected to two-way ANOVA in a factorial design (3 levels of duckweed density × 5 levels of effluent concentration) using GenStat® 17th Edition statistical software (VSN International, 2014). Data from the pot trial were subjected to analysis of variance (ANOVA) using the repeated measures to assess the effects of nutrient sources on dry matter yield, tissue nutrient composition of the ryegrass at each cut, soil nutrients and pH after harvest. Treatment means were separated using Tukey’s test at the 5% level of significance.

## Results

### Nitrogen removal by duckweed biomass and effects on solution composition

**Fig. 1:**
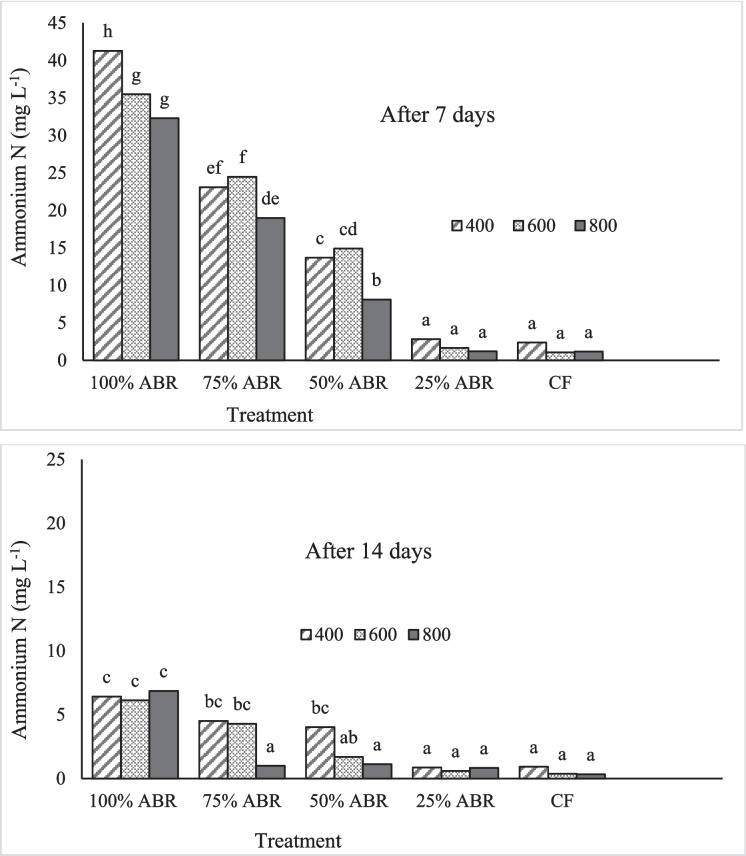
Solution ammonium-N in mg L^−1^ as affected by effluent dilution and duckweed density. Means with different letters differ significantly in each column for each period. CF = chemical fertiliser solution; 100% ABR, 75% ABR, 50% ABR, 25% ABR = effluent: tap water ratios of 1:0 (undiluted); 3:1, 1:1, and 1:3 in the mixture. Initial concentration of ammonium-N in the dilutions was 15.8–63.1 mg L^−1^ as shown in Table 1. South African threshold = 3 mg ammonium-N L.^−1^ (Department of Water Affairs, 2013)

There were significant interaction effects of duckweed density and effluent dilution on ammonium-N after both 7 and 14 days (*p* < 0.05). After 7 days, ammonium-N in the solution was in the order: undiluted effluent > 3:1 > 1:1 > 1:3 = mineral fertiliser (CF) (Fig. 1). The ammonium-N after 14 days was in the order: undiluted effluent > 3:1 = 1:1 > 1:3 = mineral fertiliser at 400, ABR effluent = 3:1 > 1:1 = 1:3 = CF at 600 g m^−2^ inoculation density, while at 800 g m^−2^, the undiluted effluent had higher concentrations than the other treatments (Fig. 1). Only the 1:3 dilution and mineral fertiliser (CF) had < 3 mg L^−1^ of ammonium-N at all duckweed densities, while for the 3:1 (18.9–25.5 mg L^−1^) and 1:1 (8.1–14.9 mg L^−1^) the concentrations in the 800 g m^−2^ were lower than the other two densities after 7 days (Fig. 1). After 14 days, the ammonium-N was < 3 mg L^−1^ in the mineral fertiliser and 1:3 dilution at all duckweed densities, and for 1:1 dilution at duckweed densities of 600 and 800 g m^−2^ (Fig. 1). The nitrate–N concentration was ≤ 3.0 mg L^−1^ for all the treatment combinations. With reference to initial concentrations, the ranges of ammonium-N removal percentages in the undiluted effluent, 3:1, 1:1, 1:3, and CF were 35–48, 48–60, 53–74, 82–92, and 73–89%, respectively, after 7 days and 89–90, 91–98, 87–96, 95–96, and 90–96%, respectively, after 14 days, with greater percentage removal at higher duckweed density.

### Duckweed biomass, growth rate, and tissue nitrogen and phosphorus

The interactions of dilution of ABR effluent and inoculation density did not significantly (*p* > 0.05) affect dry matter of duckweed. The dry matter was not significantly affected by the density of inoculation with duckweed (Table 2). The average growth rate was 1.51, 2.11, and 1.69 g m^−2^ day^−1^ for the 400, 600, and 800 g m^−2^ densities, respectively (Table 2). Dry matter was significantly lower in the ABR effluent and 3:1 (ABR effluent: water) ratio than in 1:1, 1:3, and the CF (Table 2). The growth rate averaged 0.37, 1.16, 2.33, 2.55, and 2.44 g m^−2^ day^−1^ for ABR effluent, 3:1, 1:1, 1:3, and CF, respectively (Table 2). An increase in duckweed root growth was observed with increased dilution of ABR effluent. Tissue N in duckweed was lower at 400 g m^−2^ than at the other two densities, while only the undiluted effluent had lower values than the dilutions, which had higher values than the CF treatment (Table 2). Duckweed tissue P was lower in the 400 g m^−2^ than in the 800 g m^−2^ density, and similar to the removal rate in the dilutions of the ABR (i.e. 3:1 > ABR > 1:1 > CF > 1:3) (Table 2).

**Table 2:** Dry matter, growth rate, and tissue nitrogen and phosphorus of duckweed as affected by inoculation density and ABR dilutions after 14 days

ABR dilutions	Dry matter (g m^−2^)	Average growth rate (g m^−2^ day^−1^)	Tissue N (%)	Tissue P (%)
Inoculation density				
400	21.1a	1.50a	2.71 a	0.383a
600	29.5a	2.37b	2.86 b	0.405ab
800	23.7a	1.98ab	2.96 b	0.425b
ABR dilutions				
CF	34.1b	2.45b	2.55a	0.386b
ABR	5.14a	0.66a	2.77b	0.461c
3:1	16.2a	1.38a	2.97c	0.457c
1:1	32.7b	2.51b	3.06c	0.411b
1:3	35.7b	2.76b	2.88bc	0.307a

Means within each factor in each column (parameter) with different letters are significantly different. *CF*, hydroponic solution; *ABR*, undiluted effluent; 3:1, 1:1, and 1:3 are ratios of effluent tap water

### Ryegrass dry matter yield and nutrient uptake and selected soil properties at harvest of the crop fertilised with duckweed biomass as a nitrogen and phosphorus source

**Fig. 2:**
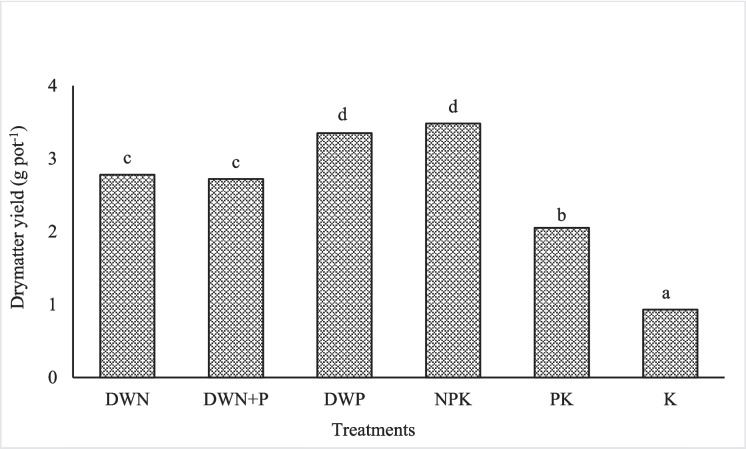
Ryegrass dry matter, nitrogen, and phosphorus uptake and extractable soil P after harvest. DWN, duckweed as an N-source; DWP, duckweed as a P-source; DWN + P, duckweed as an N-source + mineral P; NPK, positive control (mineral fertiliser); PK, negative control (without N); K, negative control (without N and P). **`** Means followed by different superscript letter(s) are significantly different

Cumulative ryegrass DM yield was higher in the NPK treatment (3.48 g pot^−1^) and when duckweed biomass was applied as a P-source (DWP) (3.2 g pot^−1^) than as an N-source (DWN) (2.5 g pot^−1^) (Fig. 2). Additional P in the DWN + P treatment did not significantly increase the DM when compared to the DWN (Fig. 2). Dry matter yield from the duckweed treatments was significantly higher than those observed from the controls, PK, and K treatments, where the addition of P increased dry matter from 0.98 to 2.05 g pot^−1^. The K treatment had significantly lower N uptake than the DWP (duckweed as a P-source) and NPK (inorganic fertiliser) treatments, while all other treatments were similar. There were no significant differences in P uptake between the duckweed treatments, which had higher values than the K treatment (Table 3). The DWN + P and the NPK treatments had higher P uptake than the PK and K treatments (Table 3). Of the duckweed treatments, only the DWP had higher extractable soil P than the K treatment, while NPK and PK treatments had significantly higher values than the duckweed treatments (Table 3).

**Table 3:** Ryegrass dry matter, nitrogen, and phosphorus uptake and extractable soil *P* after harvest

Nutrient source	Nutrient uptake (mg pot^−1^)		Extractable soil *P* (mg kg^−1^)
	N	P	
DWN	‘84.2ab	7.7bc	9.81ab
DWP	106.1b	10.4bc	12.31b
DWN + P	87.6ab	12.3c	9.47ab
NPK	115.1b	21.4c	49.78d
PK	65.1ab	6.2ab	20.64c
K	39.1a	2.1a	4.23a

*DWN*, duckweed as *N* source; *DWP*, duckweed as *P* source; DWN + P, duckweed as *N* source + mineral *P*; *NPK*, positive control (mineral fertiliser); *PK*, negative control (without N); *K*, negative control (without *N* and *P*). ‘ means followed by different superscript letter(s) in each column, are significantly different

## Discussion

### Nitrogen removal by duckweed biomass and effects on solution composition

The concentrations of N in the effluent, after both 7 and 14 days, were explained by initial concentrations and duckweed density. The generally higher percentage N removal (> 80%) in the 1:3 ratio and CF than the other solutions was a result of lower initial concentrations (< 20 mg L^−1^) (Table 1), and as such, even at the lowest (400 g m^−2^) density, threshold ammonium-N concentrations for disposal (< 3 mg L^−1^) were reached within 7 days. While the ammonium-N removal rates were lower in the 1:1 and 1:3 effluent: water ratios, and CF, the lower initial concentrations made it possible to reach the disposal threshold. The significantly higher ammonium-N at lower (400 g m^−2^) than the higher (up to 800 g m^−2^) duckweed density, in the raw ABR, 3:1, and 1:1 effluent: water ratios could be explained by a lower removal percentage. Even though the removal percentage was higher than the other solutions, and over 90% ammonium-N was removed from the raw ABR and the 3:1 effluent: water ratio, the final solutions (after 14 days) did not meet the South African disposal threshold of 3 mg L^−1^ (Department of Water Affairs, 2013), except for the 3:1 ratio with 800 g m^−2^ density, due to the higher initial concentrations (Table 1). The percentages of ammonium-N removal of 78–89, 86–98, 83–96, and 95–96% in raw ABR, 75 (3:1), 50 (1:1), and 25% (1:3) dilutions of ABR effluent, respectively, were similar to the findings of Nhapi (2004) for a variety of duckweed species, and Verma and Suthar (2014) with *L. gibba*, both on wastewater. The slight increase in nitrate–N up to 5 mg L^−1^ in the raw ABR effluent only, which was lower than the 15 mg L^−1^ threshold (Department of Water Affairs, 2013), indicated that the reduction in ammonium-N was not due to nitrification, but was a result of uptake by duckweed fronds, as biomass accumulated. It is essential to note that the initial concentration of orthophosphate P in the raw ABR effluent (11.5 mg P L^−1^) was only slightly higher than the threshold for disposal (10 mg L^−1^), as stipulated by the Department of Water Affairs South Africa (Department of Water Affairs 2013).

The removal of ammonium-N by duckweed explained the reduction of EC in the ABR solutions. The EC values for all other solutions were at or below the 1.0 dS m^−1^ threshold for safe disposal in water bodies (Department of Water Affairs, 2013), after 14 days. The similar pH range in the final (7.60–7.99) and initial solutions (pH 7.67–7.86) suggested that treatment with duckweed does not affect effluent pH, which was within the safe for disposal range (pH 5.5–9.5) (Department of Water Affairs, 2013). The removal of N through uptake during growth of *L. minor* was also associated with a significantly reduction in turbidity and coliform bacteria from the undiluted ABR effluent (Clack et al., 2019). The higher removal rate of N (800 g m^−2^) than the 400 gm^−2^ inoculation density was a result of a greater initial number of fronds, which reproduced and took up the nutrients.

### Duckweed biomass, growth rate, and tissue nitrogen and phosphorus

Although there were no significant differences in dry matter between the duckweed densities, the higher tissue N at the 600 than the 400 g m^−2^, with no increase at 800 g m^−2^, suggests that there is no significant benefit in using duckweed density higher than 600 g m^−2^. The lower growth rates in the undiluted effluent and the 3:1 dilution than the other dilutions (Table 2) corresponded with a higher removal percentage of ammonium-N, indicating that uptake of high levels of ammonium-N could cause toxicity, negatively affecting the growth rate (Leng, 1999). Solution ammonium-N ranging from 20 to 50 mg N L^−1^ has been reported as ideal for optimum growth of *Lemna* species (Chin et al., 2011). The 47.3 mg N L^−1^ in the 3:1 (effluent: water) ratio was at the upper limit of the optimum range, while the undiluted effluent contained 63 mg ammonium-N L^−1^. While salt concentration is known to affect duckweed growth, the EC of the undiluted effluent (1.33 dS m^−1^) and the 3:1 dilution (1.01 dS m^−1^) were not high enough to limit nutrient uptake. Iqbal et al. (2017) reported that maximum growth and N and P removal by *L. minor* grown on dumpsite leachate occurred at 1.0 dS m^−1^, with a slight decline observed at 1.5 dS m^−1^. Wendeou et al. (2013) also reported that EC > 1.2 dS cm^−1^ reduced the relative growth rate of duckweed species.

Of those treatments that had higher growth rate and dry matter than the undiluted effluent and the 3:1 dilution, the 1:1 dilution had the highest N and P removal rates, tissue N, and, to some extent, tissue P, than the more dilute one (1:3), which required double the amount of freshwater to dilute the effluent. The highest average growth rate of 2.76 g m^−2^ day^−1^ in the 1:3 mixture was lower than that reported by Muradov et al. (2014) for *Landoltia punctata* (3.0–4.9 g m^−2^ day^−1^), but was higher than that reported by Xu and Shen (2011) for *Spirodela oligorrhiza*, both with swine effluent, emphasising the importance of differences in duckweed species and effluent type. The lower growth rate in our study (*L. minor*) than that reported by Ge et al. (2012) for the same species (3.5 g m^−2^ day^−1^), grown on swine effluent, could be explained by differences in the media used. Although not measured separately, the root biomass of duckweed observed in the most dilute 1:3 dilution showed nutrient depletion, and it contributed to the higher biomass (Table 2). Studies by Ericsson et al. (1982) and Barks and Laird (2015) showed that, when nutrients are depleted, *L. minor* produces excessive roots to access nutrients (especially P) at greater depths. Considering the rapid growth rate, biomass production, N removal rates, freshwater requirements, and higher tissue N concentration, the 1:1 (effluent: water) ratio would be the best mixture for nutrient removal from ABR effluent using duckweed (*L. minor*) used in this study. The high N and P concentrations in the duckweed tissue, coupled with a low C/N of 8.8, suggest that the biomass can rapidly decompose, leading to mineralisation of the nutrients, particularly N (Chikuvire et al., 2018b). The duckweed (*L. minor*) grown on ABR effluent has potential as an organic fertiliser, especially if the coliforms it may also absorb are inactivated. Clack et al. (2019) reported that removal of coliforms from ABR effluent resulted in their accumulation in the *L. minor* biomass, which could be detected even after drying. Despite the challenge of coliform accumulation, duckweed dry matter grown on ABR effluent showed potential as a source of nutrients for ryegrass.

### Fertiliser value of duckweed biomass on ryegrass dry matter and nutrient uptake and selected soil properties

The higher dry matter yield of perennial ryegrass on soil amended with the different duckweed treatments, compared to the negative control (K treatment), was explained by increased availability of N and P upon decomposition of the duckweed residues, increasing uptake. The soil was kept at optimum moisture and temperature conditions during the experiment, which aided duckweed tissue decomposition (Baldock & Skjemstad, 1999; Lamb et al., 2014). Duckweed tissue is reported to have higher simple sugars, amino acids, and proteins, and lower cellulose and lignin, which supports rapid decomposition and release of mineral nutrients, than vascular plants (Iqbal, 1999; Leng, 1999). The soil used in this study (Catref) was excessively deficient in plant essential nutrients, particularly N and P, which are required in large quantities for optimal crop growth and yield (Rita et al., 2013). The addition of duckweed and NPK treatments increased available nutrients, which resulted in greater dry matter yield.

The lower cumulative dry matter (DM) yield of ryegrass in DWN (and DWN + P) treatments than in the DWP could be explained by greater N availability from the decomposition of duckweed applied at a higher rate. However, the lower N uptake in the pots with the duckweed treatments compared to the NPK treatment was because N in the duckweed was in organic form, and the rate of mineralisation was not high enough to make N available at the same rate as the one supplied entirely in inorganic form (NPK). The N in the DWN and DWP treatments was applied at 200 and 417 kg N ha^−1^ respectively, whereas P in the DWN and DWP was equivalent to 30 and 80 kg P ha^−1^. Although the N uptake was not different between the two treatments, the DWP had slightly higher, although it was not significant. The similarity of dry matter yield between the DWP and the NPK treatments suggests that the decomposition of duckweed in the DWP treatment mineralised sufficient N and P for ryegrass growth, to achieve a similar yield to the inorganic fertiliser. The supplementation of the duckweed biomass (DWN + P) with mineral P had no marked effects on the DM yield of perennial ryegrass when compared to the DWN. The lack of differences in ryegrass biomass between the DWN + P and the DWN treatments indicated that additional P was not required, especially for perennial ryegrass. Burkitt et al. (2010) and Findlay (2010) reported that ryegrass growth was not limited at the lowest P application levels, which suggests that the differences in dry matter were more in response to N availability than P. The higher DM in the DWP (417 kg N ha^−1^) showed that higher N than the recommended rate was required to reach a similar yield to the inorganic NPK fertiliser. This is because not all the N was mineralised in the 10 weeks (two weeks pre-incubation + 8 weeks of ryegrass growth). The higher dry matter in the DWP than the DWN + P (similar P levels) could be explained by higher N levels in the duckweed dry matter (DWP). Although N and P uptake in the PK treatment was not significantly higher than in the K treatment, the presence of P explains the higher ryegrass yield compared to the K treatment. The higher P uptake in the DWN + P and NPK than the PK and K treatments was mainly a result of added mineral P together with sufficient N, resulting in greater biomass accumulation.

The higher soil pH in the DWP than the PK and K controls could be a result of ammonia production during decomposition of residues applied at a high N rate (Baldock & Skjemstad, 1999; Lamb et al., 2014). While nitrification could lower soil pH, that effect was not significant in this study. The higher extractable P in the DWP than the other duckweed treatments was a result of P added in the higher quantity of duckweed, which decomposed and resulted in higher P mineralisation, while uptake by perennial ryegrass was the same. Conversely, the lower extractable P in the DWN + P than the PK treatment can be explained by higher P uptake in the DWN + P, due to higher availability and uptake of N and higher DM. The poor growth of ryegrass observed in the PK treatment could explain the poor N uptake, resulting in higher P retained in the soil. The K treatment did not get any N or P treatment and, as a result, it had the lowest DM yield and soil residual P, because none was added to the poor-quality soil.

## Conclusion

The study aimed to assess the effects of duckweed inoculation densities and dilutions of ABR effluent on biomass accumulation, uptake of N and P by common duckweed, and the quality of the residual water. The N and P fertiliser value of the duckweed (*L. minor*) biomass was also tested with perennial ryegrass. The higher growth rate and tissue N at the 600 than the 400 g m^−2^, while the growth rate and tissue N were not higher at 800 g m^−2^, indicated that 600 g m^−2^ is the most appropriate density to use when removing N and P from ABR effluent. Considering the rapid growth rate, biomass production, N and P removal, and freshwater requirements for dilution, the 1:1 effluent: water would be the best mixture for nutrient removal from ABR effluent using *L. minor*. It can, therefore, be concluded that the best combination is dilution of ABR effluent to 50% (1:1 ratio) of its original concentration and treatment with duckweed (*L. minor*) at a density of 600 g m^−2^, to reduce ammonium-N in the effluent to a quality suitable for disposal to less than the disposal threshold in 14 days. Where freshwater is limited, the 3:1 ratio can be used, with the L. minor applied at 800 g m^−2^.

Duckweed biomass, grown at a density of 600 g m^−2^, on 1:1 (ABR effluent: water) ratio, increased ryegrass dry matter and uptake of N and P when compared to the PK and K controls, particularly when added at high levels as a P source. When applied at a rate to supply enough P (DWP), duckweed biomass resulted in similar ryegrass dry matter as for the NPK fertiliser, which was higher than when the duckweed was applied to supply just enough N, based on tissue N concentration. The higher extractable P in soils amended with duckweed, can be beneficial for subsequent crops. While the N fertiliser value was significant, further studies should focus on the treatment to sterilise the duckweed of the *E. coli* which may accumulate in the biomass when grown on effluent, if the material is to be used as a safe organic fertiliser material. Other further experiments should be conducted on the possible use of duckweed biomass as an organic nutrient source on a range of crop species and soil types, under field conditions. The results of such studies will guide the formulation of policy to use duckweed to recover nutrients from wastewaters and to use the biomass as an organic nutrient source for crop productivity.

## Data Availability

No datasets were generated or analysed during the current study.
